# Thigh muscle mass evaluated by point-of-care ultrasound is associated with short-term mortality in patients with sepsis in the emergency department

**DOI:** 10.1038/s41598-024-63769-3

**Published:** 2024-06-04

**Authors:** Sejoong Ahn, Bo-Yeong Jin, Jong-Hak Park, Sungjin Kim, Sukyo Lee, Sungwoo Moon, Hanjin Cho

**Affiliations:** 1 Department of Emergency Medicine, Korea University Ansan Hospital, 123, Jeokgeum-ro, Danwon-gu, Ansan-si, Gyeonggi-do 15355 Republic of Korea; 2 Department of Biomedical Sciences, Seoul National University College of Medicine, Seoul, Republic of Korea

**Keywords:** Sonography, Muscle, Quadriceps femoris, Emergency department, Sepsis, Survival, Infectious diseases, Medical research, Infection, Risk factors

## Abstract

Muscle mass depletion is associated with mortality and morbidity in various conditions including sepsis. However, few studies have evaluated muscle mass using point-of-care ultrasound in patients with sepsis. This study aimed to evaluate the association between thigh muscle mass, evaluated using point-of-care ultrasound with panoramic view in patients with sepsis in the emergency department, and mortality. From March 2021 to October 2022, this prospective observational study used sepsis registry. Adult patients who were diagnosed with sepsis at the emergency department and who underwent point-of-care ultrasounds for lower extremities were included. The thigh muscle mass was evaluated by the cross-sectional area of the quadriceps femoris (CSA-QF) on point-of-care ultrasound using panoramic view. The primary outcome was 28 day mortality. Multivariable Cox proportional hazard model was performed. Of 112 included patients with sepsis, mean CSA-QF was significantly lower in the non-surviving group than surviving group (49.6 [34.3–56.5] vs. 63.2 [46.9–79.6] cm^2^, p = 0.002). Each cm^2^ increase of mean CSA-QF was independently associated with decreased 28 day mortality (adjusted hazard ratio 0.961, 95% CI 0.928–0.995, p = 0.026) after adjustment for potential confounders. The result of other measurements of CSA-QF were similar. The muscle mass of the quadriceps femoris evaluated using point-of-care ultrasound with panoramic view was associated with mortality in patients with sepsis. It might be a promising tool for determining risk factors for mortality in sepsis patients in the early stages of emergency department.

## Introduction

Sepsis is a life-threatening organ dysfunction caused by a dysregulated host response to infection^1,2^. Although mortality has decreased over the decades, sepsis remains a major global burden with high mortality^3,4^. Additionally, organ dysfunction is more prevalent and severe in patient who visit emergency department (ED) due to sepsis^5^. Therefore, early identification of patients with sepsis with high mortality risk is important in the ED.

Muscle mass depletion is associated with mortality and morbidity in various conditions, such as in the elderly^6–8^, transplantation^9^, trauma^10^, and sepsis^11–13^. Previous studies have evaluated muscle mass using computed tomography (CT)^6–13^. Previous studies have measured muscle mass at the thigh muscle^6^, paravertebral muscle^7,11^, pectoralis muscle^8^, and psoas muscle^9,13^. Few studies have evaluated the muscle mass measured using ultrasonography^14,15^. Another study used arm or thigh circumference to measure muscle mass^16–18^.

Performing CT is expensive, carries the risk of radiation exposure, and requires patient transfer to CT^19^. In contrast, ultrasound is safe without radiation exposure, less expensive than CT, and can be performed at the bedside of patients^19^. Additionally, compared with CT, point-of-care ultrasound can be performed rapidly in the ED. Ultrasound has recently been considered one of diagnostic tools to evaluate muscle mass.

However, few studies have evaluated muscle mass using ultrasonography in patients with sepsis in the ED. Recently introduced point-of-care ultrasound with panoramic view allows for the examination of large muscles and can be performed rapidly and noninvasively at the bedside in the ED. Therefore, this study aimed to evaluate the association between thigh muscle mass, evaluated using point-of-care ultrasound with panoramic view in patients with sepsis in the emergency department, and mortality.

## Methods

### Study design and setting

This prospective observational study was conducted at the Korea University Ansan Hospital. The Korea University Ansan Hospital is the only tertiary academic teaching hospital in Ansan-si, where approximately 700,000 residents live. Around 50,000 patients visit the ED of Korea University Ansan Hospital annually^20^. This study was conducted in accordance with the principles of the Declaration of Helsinki. The Institutional Review Board of Korea University approved this observational study (2022AS0299) and waived the requirement for informed consent due to the nature of the study.

### Definitions and data collection

Sepsis was defined as an acute increase in the total sequential organ failure assessment (SOFA) score ≥ 2 from baseline due to infection^1^. Septic shock was defined as a serum lactate level > 2 mmol/L and requirement of vasopressor despite adequate fluid resuscitation to maintain mean arterial pressure ≥ 65 mmHg. All patients were managed according to the surviving sepsis campaign guidelines^2^.

The following patient data were extracted from electronic medical records: age, sex, height, weight, body mass index (BMI), comorbidities, infection focus, septic shock status, initial vital signs at the ED, initial SOFA score at the ED, initial laboratory and image results at the ED, vasopressor use, mechanical ventilator use, and 28 day mortality.

Comorbidities were classified as hypertension, diabetes, heart disease (congestive heart failure, prior myocardial infarction, or cardiomyopathy), lung disease (chronic obstructive pulmonary disease or interstitial primary fibrosis), liver cirrhosis, chronic kidney disease, stroke (ischemic or hemorrhagic stroke), and malignancy (hematologic malignancy or solid tumor). The infection focus was classified as respiratory, gastrointestinal, genitourinary, or other.

### Patient position and thigh muscle mass measurement

The patients were placed in a supine position on the bed and as relaxed as possible. The legs were set in an anatomical position, with the toes pointing to the ceiling. Thigh muscle mass was evaluated by the cross-sectional area of the quadriceps femoris (CSA-QF) using point-of-care ultrasound (GE Healthcare, WI, USA). CSA-QF was defined as the cross-sectional area surrounded by the outer fascia of the components of the quadriceps femoris (rectus femoris, vastus medialis, vastus intermedius, and vastus lateralis). CSA-QF was measured at the midpoint between the anterior superior iliac spine and the upper edge of the patella. CSA-QF was measured three times on each side (right and left) using the panoramic mode (Supplementary Fig. 1A–D). During the CSA-QF measurements, the pressure of the probe was minimized. CSA-QF was measured after initial resuscitation at the ED.

The mean CSA-QF, minimum CSA-QF, right mean CSA-QF, left mean CSA-QF, dominant leg mean CSA-QF, and non-dominant leg mean CSA-QF were evaluated in the analyses.

The point-of-care ultrasound with panoramic view was performed by six emergency physicians with four to more than 10 years of experience in the emergency department and point-of-care ultrasound. They had undergone several practice sessions starting one month before the study commencement and received feedback on the acquired images. During the study period, the measurements on each thigh for three times were typically completed within 5 min.

### Study population

Adult (age > 18 years) patients diagnosed with sepsis at the ED who underwent lower extremity point-of-care ultrasounds from March 2021 to October 2022 were included in this study. Patients with deformities in the lower extremities, who were unable to maintain the appropriate position for thigh muscle mass measurement, and who had a do-not-resuscitate order were excluded.

### Sample size calculation

The previous study, which evaluated the association between muscle thickness evaluated by ultrasound and mortality, included 70 patients based solely on feasibility^14^. This study aimed to analyze 100 patients as a pilot study to evaluate the association between thigh muscle mass evaluated by the novel method of point-of-care ultrasound with panoramic view and mortality. Considering that 1/3 of patients would be excluded due to abovementioned criteria, 150 patients were planned to undergo point-of-care ultrasound on lower extremities.

### Outcomes

The primary outcome was the 28 day mortality. The secondary outcomes were 7 day mortality, 14 day mortality, and mechanical ventilator use within 24 h.

### Statistical analysis

Continuous variables with normal distribution were expressed as means ± standard deviations and compared using student’s *t*-test. Continuous variables without a normal distribution were expressed as medians [interquartile ranges] and compared using the Mann–Whitney test. Categorical variables are expressed as numbers and percentages and compared using the chi-square or Fisher’s exact test.

To evaluate factors associated with mean CSA-QF, multivariable linear regression analysis was performed. The factors that were significant at a level of 0.1 in the univariable model were entered into multivariable linear regression model.

After adjusting for confounders, multivariable Cox proportional hazard model was used to determine an independent association between thigh muscle mass and 28 day mortality. The variables that were significant at a level of 0.1 in the univariable model and those selected by the researchers (age, sex, BMI, septic shock status, SOFA score, lactate) were used in the multivariable Cox proportional hazard model. Additionally, stepwise backward elimination multivariable Cox proportional hazard model was performed.

The receiver operating characteristic curve of CSA-QF for 28 day mortality was performed. The optimal cutoff points were obtained using the Youden index. Primary and secondary outcomes were evaluated according to the optimal cutoff points. Kaplan–Meier curves and log-rank tests were performed according to the optimal cutoff points.

Subgroup analysis was performed in patients with septic shock.

The level of significance was set at p < 0.05. All Statistical analyses were performed using R version 4.0.2 (R Foundation for Statistical Computing, Vienna, Austria).

### Ethics approval

The Institutional Review Board approved this study (2022AS0299) and waived the requirement for informed consent due to the nature of the study.

## Results

**Figure 1:**
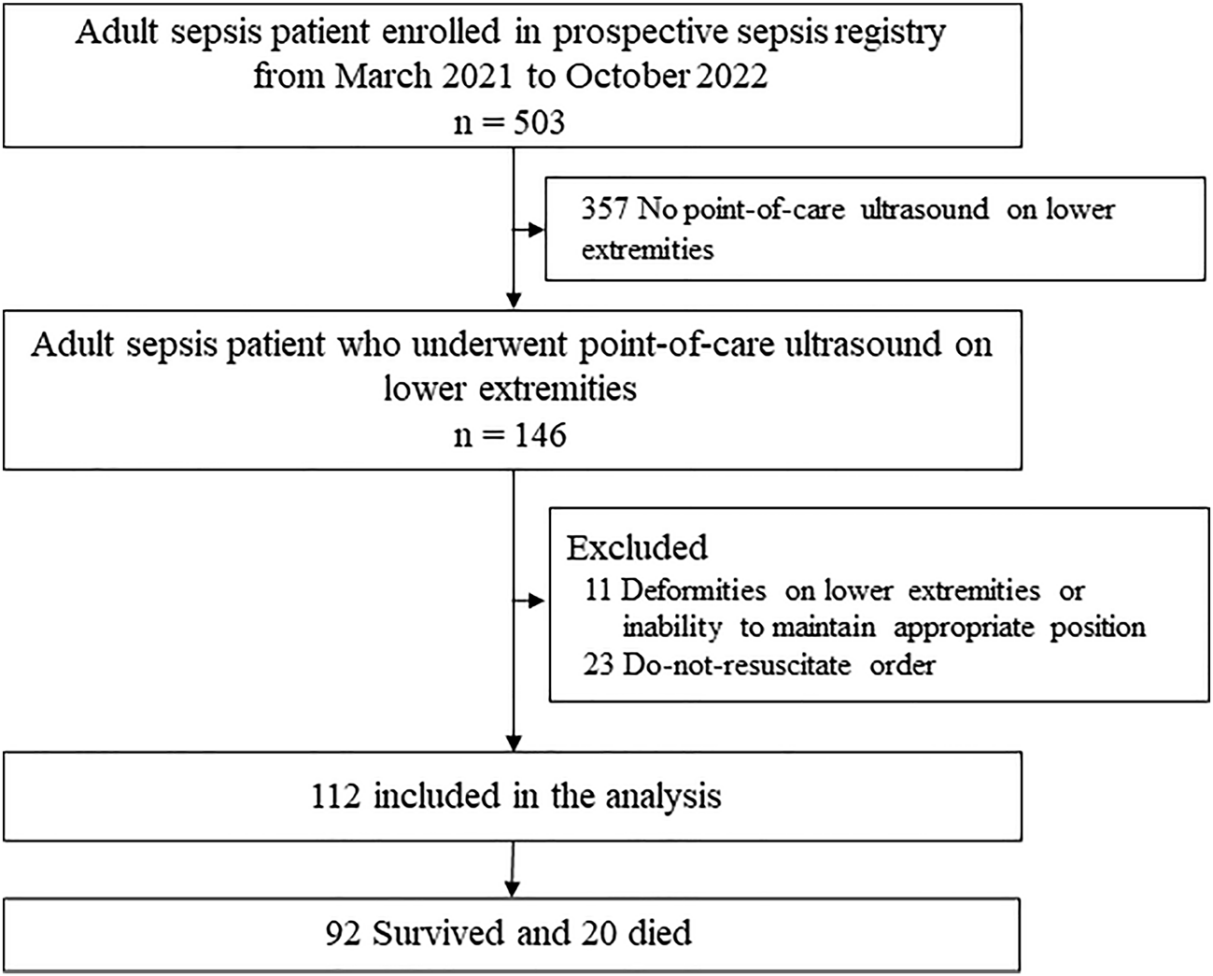
Flow diagram of the study population.

From March 2021 to October 2022, a total of 503 adult patients were enrolled in prospective sepsis registry. Among them, 146 patients underwent point-of-care ultrasounds of the lower extremities. Eleven patients were excluded because of deformities in the lower extremities or an inability to maintain an appropriate position for thigh muscle mass measurement. Twenty-three patients were excluded because of a do-not-resuscitate order. Finally, 112 patients were included in this study (Fig. 1). Of the 112 patients, 64 (57.1%) were male, and the mean age was 70.6 ± 14.4 years. The mean SOFA score was 6.4 ± 3.3, and 20 (17.9%) patients died on day 28.

Table 1 shows baseline demographics and characteristics of study populations. The mean CSA-QF was significantly lower in the non-surviving group than in the surviving group (49.6 [34.3–56.5] vs. 63.2 [46.9–79.6] cm^2^, p = 0.002). All other measurements of CSA-QF were significantly lower in the non-surviving group than surviving group (Table 1).

**Table 1:** Demographics and characteristics of the study population.

Variables	Survived at day 28 (n = 92)	Died (n = 20)	p-value
Age (years)	73 [60–81.5]	78 [65–85]	0.438
Sex			
Men	55 (59.8%)	9 (45.0%)	0.336
Women	37 (40.2%)	11 (55.0%)	
BMI (kg/m^2^)	24.1 ± 4.5	22.3 ± 4.4	0.108
Comorbidities			
Hypertension	45 (48.9%)	12 (60.0%)	0.514
Diabetes mellitus	29 (31.5%)	12 (60.0%)	0.032
Liver disease	9 (9.8%)	0 (0.0%)	0.315
Heart disease	14 (15.2%)	4 (20.0%)	0.848
Cerebrovascular disease	20 (21.7%)	3 (15.0%)	0.711
Lung disease	8 (8.7%)	4 (20.0%)	0.279
Chronic kidney disease	11 (12.0%)	5 (25.0%)	0.247
Malignancy	20 (21.7%)	5 (25.0%)	0.983
Initial vital signs			
Systolic blood pressure (mmHg)	112.5 [96–134]	111 [96.5–139]	0.970
Diastolic blood pressure (mmHg)	63 [53.0–76.5]	62.5 [57–77]	0.712
Heart rate (/min)	105.5 ± 27.1	106.0 ± 22.3	0.931
Respiratory rate (/min)	20 [18–24]	24 [20–30]	0.010
Body temperature (℃)	37.2 [36.7–38.4]	36.8 [36.1–38.1]	0.142
SpO_2_ (%)	96.5 [95–98]	96.0 [92–98]	0.371
Clinical data			
Infection focus			0.294
Respiratory	33 (35.9%)	11 (55.0%)	
Gastrointestinal	28 (30.4%)	4 (20.0%)	
Genitourinary	21 (22.8%)	2 (10.0%)	
Others	10 (10.9%)	3 (15.0%)	
SOFA score	5 [3–8]	8 [6.5–10.5]	0.004
Septic shock	34 (37.4%)	15 (75.0%)	0.005
Mechanical ventilator use	11 (12.0%)	13 (65.0%)	< 0.001
ICU admission	22 (23.9%)	12 (60.0%)	0.004
Laboratory data			
Lactate (mmol/L)	2.4 [1.6–3.8]	5.6 [3.1–8.1]	< 0.001
White blood cell count (*10^3^/μL)	10.0 [7.0–16.2]	13.6 [8.1–24.0]	0.180
Platelets (*10^3^/μL)	162.0 [121.0–227.0]	181.0 [118.5–267.5]	0.416
Glucose (mg/dL)	136 [111–174]	184 [116–315.5]	0.083
Creatinine (mg/dL)	1.4 [1.0–1.9]	2.4 [1.3–3.5]	0.031
Total bilirubin (mg/dL)	0.8 [0.5–1.6]	0.7 [0.4–0.9]	0.092
CRP (mg/dL)	11.4 [5.0–17.7]	12.5 [8.9–18.8]	0.386
Procalcitonin (ng/mL)	3.6 [0.7–14.4]	7.1 [0.6–29.3]	0.432
Albumin (g/dL)	3.4 ± 0.6	2.9 ± 0.7	0.002
Muscle mass of quadriceps femoris			
Mean CSA-QF (cm^2^)	63.2 [46.9–79.6]	49.6 [34.3–56.5]	0.002
Mean right CSA-QF (cm^2^)	62.9 [48.2–79.8]	49.7 [32.5–56.3]	0.001
Mean left CSA-QF (cm^2^)	62.2 [44.7–78.6]	49.4 [35.3–58.5]	0.004
Minimum CSA-QF (cm^2^)	54.3 [42.0–75.7]	45.4 [30.1–51.9]	0.001
Maximum CSA-QF (cm^2^)	68.3 [50.0–83.2]	53.8 [41.2–60.0]	0.003
Mean dominant leg CSA-QF (cm^2^)	63.6 [48.2–81.1]	49.7 [36.2–56.3]	0.001
Mean non-dominant leg CSA-QF (cm^2^)	60.0 [44.7–78.3]	49.4 [32.0–58.5]	0.004

Continuous variables with normal distribution were expressed as means ± standard deviations and compared using student’s *t*-test. Continuous variables without a normal distribution were expressed as medians [and interquartile ranges] and compared using the Mann–Whitney test. Categorical variables are expressed as numbers (percentages) and compared using the chi-square or Fisher’s exact test.

*BMI* body mass index, *SpO*_*2*_ peripheral oxygen saturation, *SOFA* sequential organ failure assessment, *ICU* intensive care unit, *CRP* C-reactive protein, *CSA-QF* cross-sectional area of the quadriceps femoris.

### Factors associated with mean CSA-QF

In multivariable linear regression model, women, old age, diabetes mellitus, and high SOFA score were independently associated with decreased mean CSA-QF (Supplementary Table 1). However, albumin was not significantly associated with mean CSA-QF (p = 0.339).

### Multivariable Cox proportional hazard model

Each cm^2^ increase in the mean CSA-QF was independently associated with decreased 28 day mortality (adjusted hazard ratio (aHR) 0.967, 95% confidence interval (CI), 0.939–0.996, p = 0.026) after adjustment (Table 2 and Supplementary Table 2). The mean CSA-QF was independently associated with decreased 28 day mortality (aHR 0.964, 95% CI 0.937–0.992, p = 0.011) in the stepwise backward elimination model. All other measurements of CSA-QF were independently associated with decreased 28 day mortality in both models (Table 2). The results of multivariable Cox proportional hazard model, which were analyzed by CSA-QFs per 10 cm^2^, are shown in Supplementary Table 3. Each 10 cm^2^ increase in the mean CSA-QF was independently associated with decreased 28 day mortality (aHR 0.716, 95% CI 0.534–0.961 in multivariable model and aHR 0.691, 95% CI 0.520–0.919 in stepwise backward elimination model, Supplementary Table 3).

**Table 2:** The result of the multivariable Cox proportional hazard model.

Variable of interest	Multivariable Cox proportional hazard model			Stepwise backward elimination model		
	aHR	95% CI	p-value	aHR	95% CI	p-value
Mean CSA-QF (cm^2^)	0.967	0.939–0.996	0.026	0.964	0.937–0.992	0.011
Mean right CSA-QF (cm^2^)	0.968	0.941–0.995	0.021	0.966	0.940–0.992	0.011
Mean left CSA-QF (cm^2^)	0.968	0.939–0.998	0.036	0.963	0.936–0.992	0.013
Minimum CSA-QF (cm^2^)	0.967	0.937–0.997	0.033	0.963	0.934–0.993	0.015
Maximum CSA-QF (cm^2^)	0.969	0.943–0.996	0.025	0.966	0.940–0.992	0.010
Mean dominant CSA-QF (cm^2^)	0.967	0.941–0.995	0.022	0.966	0.940–0.992	0.012
Mean non-dominant CSA-QF (cm^2^)	0.968	0.940–0.997	0.035	0.963	0.936–0.992	0.012

Age, sex, septic shock status, BMI, DM, lactate, glucose, albumin, and SOFA score were adjusted in the multivariable Cox proportional hazard model.

*aHR* adjusted hazard ratio, *CI* confidence interval, *CSA-QF* cross-sectional area of the quadriceps femoris.

### Receiver operating characteristic curve of CSA-QF on 28 day mortality

The area under the receiver operating characteristic curve of the mean CSA-QF for 28 day mortality was 0.722 (95% CI 0.606–0.838, p < 0.001). The optimal cutoff of the mean CSA-QF was 63.1 cm^2^, with a sensitivity of 90.0% and a specificity of 51.1%. The area under the receiver operating characteristic curve of other measurements of the CSA-QF was similar. (Table 3).

**Table 3:** The result of receiver operating characteristic curve of CSA-QF on 28 day mortality.

Variable of interest	AUROC		95% CI	p-value		Cutoff	Sensitivity	Specificity
Mean CSA-QF	0.722		0.606–0.838	< 0.001	Youden index	63.1	90.0%	51.1%
					Cutoff for specificity	41.7	40.0%	80.4%
Mean right CSA-QF	0.733		0.620–0.847	< 0.001	Youden index	60.6	90.0%	55.4%
					Cutoff for specificity	44.3	40.0%	80.4%
Mean left CSA-QF	0.708		0.591–0.824	< 0.001	Youden index	65.9	95.0%	43.5%
					Cutoff for specificity	41.8	45.0%	80.4%
Minimum CSA-QF	0.731		0.617–0.846	< 0.001	Youden index	52.5	85.0%	57.6%
					Cutoff for specificity	37.4	45.0%	80.4%
Maximum CSA-QF	0.712		0.596–0.828	< 0.001	Youden index	60.8	80.0%	60.9%
					Cutoff for specificity	47.0	40.0%	80.4%
Mean dominant CSA-QF	0.733		0.622–0.845	< 0.001	Youden index	60.6	90.0%	56.5%
					Cutoff for specificity	44.3	40.0%	80.4%
Mean non-dominant CSA-QF	0.708		0.588–0.827	< 0.001	Youden index	65.9	95.0%	42.4%
					Cutoff for specificity	41.8	45.0%	80.4%

*AUROC* area under receiver operating characteristic curve, *CI* confidence interval, *CSA-QF* cross-sectional area of the quadriceps femoris.

### Outcomes according to the optimal cutoff of CSA-QF

The 7 day, 14 day, and 28 day mortality, and mechanical ventilator use within 24 h were significantly higher in the group with low quadriceps femoris mass according to the optimal cutoff of the mean CSA-QF than in the group with high quadriceps femoris mass. (Supplementary Table 4).

**Figure 2:**
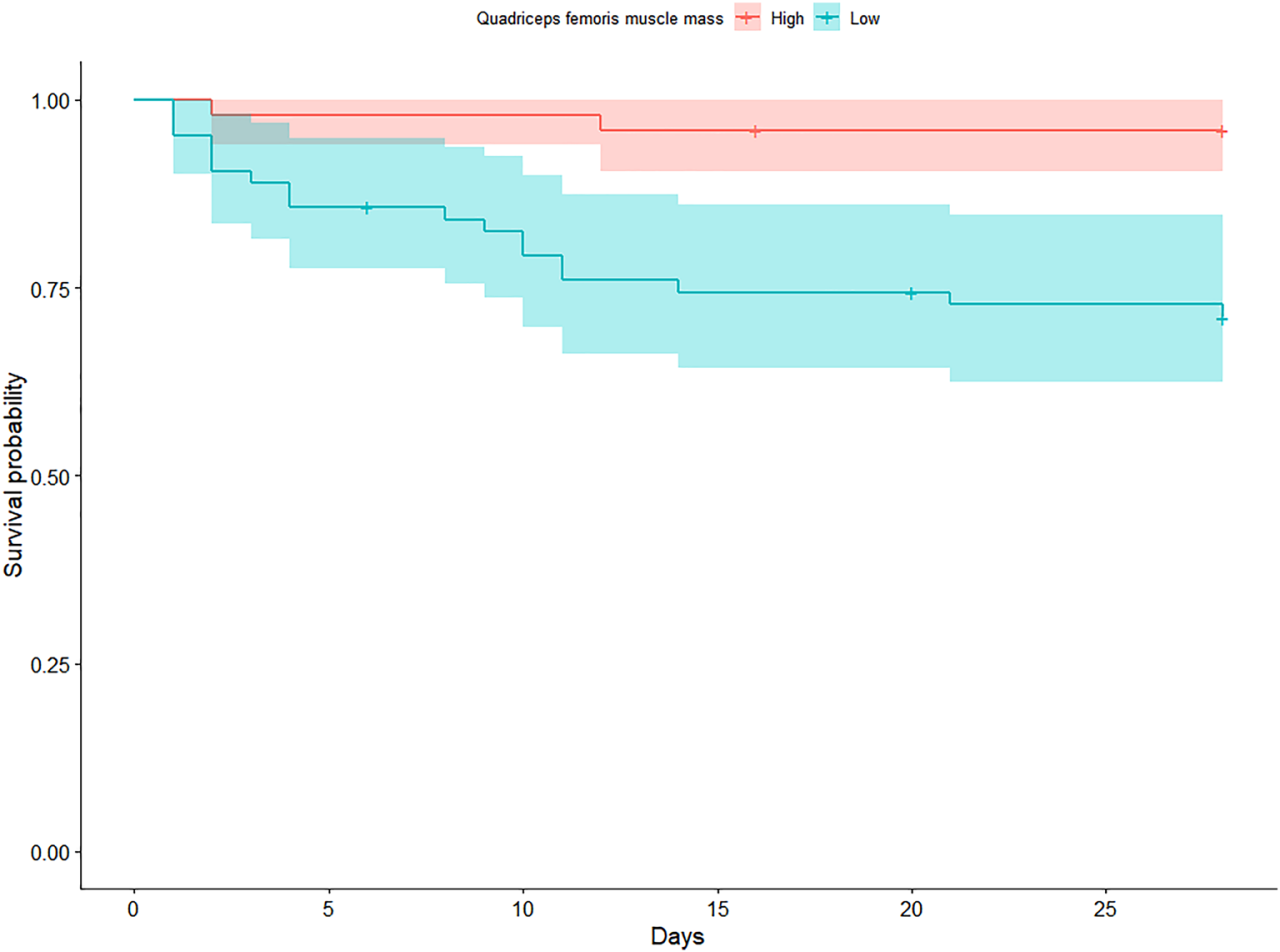
Kaplan–Meier curve for 28 day mortality according to optimal cutoff of mean CSA-QF. *CSA-QF* cross sectional area of quadriceps femoris.

Low quadriceps femoris mass according to the optimal cutoff of mean CSA-QF was associated with 28 day mortality in the Kaplan–Meier curve (log-rank test p < 0.001). (Fig. 2).

### Subgroup analysis

In the subgroup of patients with septic shock, the mean CSA-QF was significantly lower in the non-surviving group than in the surviving group (43.6 [34.3–56.4] vs. 64.3 [45.6–81.1], p = 0.005). All other measurements of CSA-QF were significantly lower in the non-surviving group than in the surviving group.

The mean CSA-QF was independently associated with a decreased 28 day mortality in patients with septic shock (aHR 0.959, 95% CI, 0.924–0.994; p = 0.023).

## Discussion

In our study, we found that the muscle mass of the quadriceps femoris evaluated using point-of-care ultrasound was independently associated with 28 day mortality in adult patients with sepsis in the ED. The mean CSA-QF was significantly lower in the non-surviving group than in the surviving group. The area under the receiver operating characteristic curve of the mean CSA-QF on 28 day mortality showed fair performance. Similar results were obtained between the CSA-QF measurement methods. According to the optimal cutoff, low quadriceps femoris mass was associated with short-term mortality and mechanical ventilator use within 24 h.

Magnetic resonance imaging (MRI) or CT is considered the gold standard for measuring muscle mass^21^. Compared to MRI and CT, ultrasonography has the advantages of low cost, relatively short time to image acquisition, no risk of complication from contrast if used, and being performed at the bedside^19,21^. Ultrasound shows accurate performance in measuring muscle mass^21^. To evaluate large muscle, the panoramic view of ultrasound can be used^22–25^. The panoramic view showed excellent inter-rater reliability and good correlation with MRI in measuring the cross-sectional areas of the quadriceps femoris (concordance correlation coefficient 0.78, p < 0.001)^22^, vastus medialis (interclass correlation coefficient about 0.98 and interrater reliability about 0.99)^23^, and hamstring muscles (correlation coefficient r ≥ 0.8)^24^. Thus, panoramic mode is a good tool for evaluating large muscles, such as the quadriceps femoris.

Low muscle mass is associated with mortality in patients with sepsis. Most previous studies evaluated muscle mass using CT, and muscle mass was calculated as either the paravertebral muscle at the L1 to L3 level or the psoas muscle^11–13^. Other previous studies measured muscle mass using arm circumferences^16^. Only a few studies have measured muscle mass using ultrasonography in the intensive care unit^14,15^. Ultrasonography can be easily performed at the bedside in the ED or intensive care unit. We measured muscle mass using point-of-care ultrasound in the ED and found that low quadriceps femoris mass was associated with mortality in patients with sepsis. We measured the quadriceps femoris in the supine position, a common position in patients with sepsis in the ED. Furthermore, measurement of the quadriceps femoris, the anterior component of the thigh muscle, can be approached easily with minimal adjustment of the patient’s or practitioner’s position. Therefore, point-of-care ultrasound can be easily, rapidly, and non-invasively performed at the quadriceps femoris. CSA-QF might be a promising tool for determining the risk factors for mortality in patients with sepsis in the early stages and at the bedside of the ED and intensive care unit.

There are limited studies on defining the normal value of quadriceps femoris, especially in patients with sepsis or critically ill disease. Based on receiver operating characteristic curve for 28 day mortality, the cutoff ranging from 60 to 65 cm^2^ showed high sensitivity, while the cutoff ranging from 40 to 45 cm^2^ showed high specificity. These cutoffs may be used as indicators of normal muscle mass or pathological muscle loss of the quadriceps in patients with sepsis. Age, sex, diabetes mellitus, and SOFA were significant factors associated with muscle mass of quadriceps femoris in linear regression model (Supplementary Table 1). These factors need to be considered when defining the normal value of quadriceps femoris muscle mass. Further research with a larger study population is needed to elucidate this issue, as this is a pilot study.

In our study, muscle mass was calculated by using the CSA-QF in the ED. In contrast, Hadda et al. evaluated thigh muscle thickness using ultrasonography in patients with sepsis in the intensive care unit^14^. Hadda et al. reported that a decline in thigh muscle thickness was associated with mortality^14^. The initial thigh muscle thickness was thicker in survivors than in non-survivors, but the difference was not statistically significant^14^. Similarly, thigh muscle thickness was not a significant factor in the present study. Since thigh muscle thickness is a single-dimensional measurement and can only measure the thickness of the rectus femoris and vastus intermedius muscles, the mass of the vastus medialis and vastus lateralis muscles cannot be evaluated^26^. Panoramic view revealed that muscle width (horizontal length) of quadriceps femoris, especially vastus medialis, vastus intermedius, and vastus lateralis, were greater than muscle thickness (vertical length) of quadriceps femoris (Supplementary Fig. 1A–D). Additionally, to evaluate muscle mass, the sum of muscle thickness at multiple sites is required^26^. For these reasons, a single measurement of thigh muscle thickness might be insufficient to reflect the true thigh muscle mass or volume, and its difference might be insufficient to reveal statistical significance. Therefore, measurement of the cross-sectional area of the quadriceps femoris, which is a two-dimensional measurement, is required to evaluate muscle mass.

Since the measurement of CSA-QF can vary, we measured CSA-QF three times and evaluated various CSA-QFs, including mean, minimum, and maximum. Further, we evaluated the combination of CSA-QF and the side of leg or leg dominance. We adjusted for potential covariables and found that all types of CSA-QF were independently associated with mortality. Thus, physicians can use any measurement of CSA-QF, regardless of leg dominance, to detect high-risk patients with sepsis in early stage of the ED.

The quadriceps femoris muscle consists of rectus femoris, vastus medialis, vastus intermedius, and vastus lateralis muscles. The vastus muscles play a more important role in knee extension and sitting-to-standing movements than the rectus femoris^26^. The component of CSA-QF differs according to the thigh location^25^. From the proximal thigh to the distal thigh, the mass of the rectus femoris and vastus intermedius decreased, and the mass of the vastus medialis increased. The muscle mass of the vastus lateralis peaked at the midpoint of the thigh^25^. Muscle wasting during bed rest was more prominent in the vastus muscles than in the rectus femoris^26^. Thus, the location of the measurement of CSA-QF is important, and the result of CSA-QF needs to be interpreted based on the patient’s condition.

Beside lactic acid and SOFA score, our findings suggest that the measurement of CSA-QF can be another method for evaluating risk factors for mortality in patients with sepsis. Even after adjusting for important variables such as lactic acid and SOFA score, the CSA-QFs were found to be independently associated with mortality. Furthermore, the performance of CSA-QF was similar to that of lactic acid and SOFA score (Supplementary Table 5). Therefore, muscle mass of quadriceps femoris can be additional prognostic factor for patients with sepsis.

In our study, albumin, a well-known nutritional factor^27^, was not associated with CSA-QF. Other well-known nutritional factors include total lymphocyte count, nitrogen, and creatinine^28,29^. Even when other well-known nutritional factors^27–29^ were additionally analyzed, these nutritional factors were not significantly associated with CSA-QF (Supplementary Table 6). Measuring muscle mass might be another tool to evaluate nutritional status of the patient. Further studies are warranted on muscle mass as a nutritional factor.

Prolonged immobilization, poor nutrition, and a hypercatabolic state significantly decrease the muscle mass. A previous study reported that 2% of skeletal muscles were wasted each day during the acute phase of critically ill patients^30^. The muscle mass may indicate overall consequential conditions of a critically ill state, age, comorbidities, previous prolonged immobilization, poor nutrition, and hypercatabolic state. The advantage of point-of-care ultrasound of quadriceps femoris can be more valuable, especially in patients with unknown previous medical history or conditions.

Low quadriceps femoris muscle mass was associated with mechanical ventilator use within 24 h. Previous studies reported that low muscle mass was associated with prolonged mechanical ventilator use^7,8,15,31^. Emergency physicians should be aware of the high risk of mechanical ventilator use and prolonged mechanical ventilator use in patients with sepsis with low muscle mass.

There are some limitations in this study. First, owing to the nature of the observational study, we could only find associations, and there could be missed variables. Second, this study was conducted at a single center and included a small number of patients with sepsis. Further large, multicenter studies are warranted. Third, the study was conducted in an Asian country. The results cannot be generalized to the entire population. Further validation is needed for other races and ethnicities. Fourth, the position of the patient might have influenced the CSA-QF measurement. Although we tried to standardize the position of the patient, which can easily be achieved in the ED, there can be measurement errors. We measured the CSA-QF multiple times to minimize error and evaluated various types of CSA-QFs. We found that all types of CSA-QFs are helpful. Fifth, we did not evaluate the decline of thigh muscle over time and muscle quality, including myosteatosis^32^. Further studies on the usefulness of ultrasonography in assessing changes in muscle mass or muscle quality are needed. Sixth, this study did not aim to evaluate the variability in measurements. Although the previous studies reported that the panoramic view showed excellent inter-rater reliability and good correlation with MRI in measuring the cross-sectional areas of the quadriceps femoris^22^, vastus medialis^23^, and hamstring muscles^24^, further studies are needed to evaluate the variability in measurements in patients with sepsis.

## Conclusions

The muscle mass of the quadriceps femoris evaluated using point-of-care ultrasound with panoramic view was associated with mortality in patients with sepsis. It can be easily measured at bedside and might be a promising tool for determining risk factors for mortality in sepsis patients in the early stages of emergency department.

### Supplementary Information

Supplementary Information.

## Data Availability

The data generated and/or analysed during the current study are not publicly available due to their containing information that could compromise the privacy of research participants but are available from the corresponding author on reasonable request.

## Abbreviations

ED: Emergency department
CT: Computed tomography
SOFA: Sequential organ failure assessment
BMI: Body mass index
CSA-QF: Cross-sectional area of the quadriceps femoris
ahr: Adjusted hazard ratio
CI: Confidence interval
MRI: Magnetic resonance imaging

## References

[1] Singer M, Deutschman CS, Seymour CW (2016). The third international consensus definitions for sepsis and septic shock (sepsis-3). JAMA.

[2] Evans L, Rhodes A, Alhazzani W (2021). Executive summary: Surviving sepsis campaign: International guidelines for the management of sepsis and septic shock 2021. Crit. Care Med..

[3] Reinhart K, Daniels R, Kissoon N (2017). Recognizing sepsis as a global health priority—A WHO resolution. N. Engl. J. Med..

[4] Font MD, Thyagarajan B, Khanna AK (2020). Sepsis and septic shock—Basics of diagnosis, pathophysiology, and clinical decision making. Med. Clin. N. Am..

[5] Yu CW, Chang SS, Lai CC (2019). Epidemiology of emergency department sepsis: A national cohort study between 2001 and 2012. Shock.

[6] Fukasawa H, Kaneko M, Niwa H (2017). Lower thigh muscle mass is associated with all-cause and cardiovascular mortality in elderly hemodialysis patients. Eur. J. Clin. Nutr..

[7] Moisey LL, Mourtzakis M, Cotton BA (2013). Skeletal muscle predicts ventilator-free days, ICU-free days, and mortality in elderly ICU patients. Crit. Care.

[8] Moon SW, Kim SY, Choi JS (2021). Thoracic skeletal muscle quantification using computed tomography and prognosis of elderly ICU patients. Sci. Rep..

[9] Bibas L, Saleh E, Al-Kharji S (2018). Muscle mass and mortality after cardiac transplantation. Transplantation.

[10] Xi F, Tan S, Gao T (2021). Low skeletal muscle mass predicts poor clinical outcomes in patients with abdominal trauma. Nutrition.

[11] Shibahashi K, Sugiyama K, Kashiura M (2017). Decreasing skeletal muscle as a risk factor for mortality in elderly patients with sepsis: A retrospective cohort study. J. Intensiv. Care.

[12] Zhang J, Huang Y, Chen Y (2021). Impact of muscle mass on survival in patients with sepsis: A systematic review and meta-analysis. Ann. Nutr. Metab..

[13] Lee Y, Park HK, Kim WY (2018). Muscle mass depletion associated with poor outcome of sepsis in the emergency department. Ann. Nutr. Metab..

[14] Hadda V, Kumar R, Khilnani GC (2018). Trends of loss of peripheral muscle thickness on ultrasonography and its relationship with outcomes among patients with sepsis. J. Intensiv. Care.

[15] Sklar MC, Dres M, Fan E (2020). Association of low baseline diaphragm muscle mass with prolonged mechanical ventilation and mortality among critically Ill adults. JAMA Netw. Open.

[16] Lucidi C, Lattanzi B, Di Gregorio V (2018). A low muscle mass increases mortality in compensated cirrhotic patients with sepsis. Liver Int..

[17] Kawamoto R, Kikuchi A, Akase T (2021). Thigh circumference and handgrip strength are significantly associated with all-cause mortality: Findings from a study on Japanese community-dwelling persons. Eur. Geriatr. Med..

[18] Heitmann BL, Frederiksen P (2009). Thigh circumference and risk of heart disease and premature death: Prospective cohort study. BMJ..

[19] Stringer HJ, Wilson D (2018). The role of ultrasound as a diagnostic tool for sarcopenia. J. Frailty Aging.

[20] Jin BY, Song J, Kim J (2023). Association between metformin and survival outcomes in in-hospital cardiac arrest patients with diabetes. J. Crit. Care.

[21] Cruz-Jentoft AJ, Bahat G, Bauer J (2019). Sarcopenia: Revised European consensus on definition and diagnosis. Age Ageing.

[22] Scott JM, Martin DS, Ploutz-Snyder R (2017). Panoramic ultrasound: A novel and valid tool for monitoring change in muscle mass. J. Cachexia Sarcopenia Muscle.

[23] Minnehan KS, Dexter WW, Holt CT (2023). Validation of panoramic ultrasound measurement of the cross-sectional area of the vastus medialis. J. Strength Cond. Res..

[24] Franchi MV, Fitze DP, Hanimann J (2020). Panoramic ultrasound vs. MRI for the assessment of hamstrings cross-sectional area and volume in a large athletic cohort. Sci. Rep..

[25] Kellis E, Sahinis C, Dafkou K (2021). Hamstring to quadriceps strength ratio and cross-sectional area of the quadriceps and hamstrings muscles assessed using extended field-of-view ultrasonography. Res. Sports Med..

[26] Sanada K, Kearns CF, Midorikawa T (2006). Prediction and validation of total and regional skeletal muscle mass by ultrasound in Japanese adults. Eur. J. Appl. Physiol..

[27] Keller U (2019). Nutritional laboratory markers in malnutrition. J. Clin. Med..

[28] Picó C, Serra F, Rodríguez AM (2019). Biomarkers of nutrition and health: New tools for new approaches. Nutrients.

[29] Haines RW, Zolfaghari P, Wan Y (2019). Elevated urea-to-creatinine ratio provides a biochemical signature of muscle catabolism and persistent critical illness after major trauma. Intensiv. Care Med..

[30] Fazzini B, Märkl T, Costas C (2023). The rate and assessment of muscle wasting during critical illness: A systematic review and meta-analysis. Crit. Care.

[31] Damanti S, Cristel G, Ramirez GA (2021). Influence of reduced muscle mass and quality on ventilator weaning and complications during intensive care unit stay in COVID-19 patients. Clin. Nutr..

[32] Correa-de-Araujo R, Harris-Love MO, Miljkovic I (2017). The need for standardized assessment of muscle quality in skeletal muscle function deficit and other aging-related muscle dysfunctions: A symposium report. Front. Physiol..

