# Supplementary material for: Thigh muscle mass evaluated by point-of-care ultrasound is associated with short-term mortality in patients with sepsis in the emergency department
Source: Sci Rep. 2024 Jun 4;14:12776. doi: 10.1038/s41598-024-63769-3 (PMC11150469; doi:10.1038/s41598-024-63769-3)

**Supplementary Table 1.** Factors associated with muscle mass of quadriceps femoris using linear regression model.

|  | Univariable model | | | Multivariable model | | |
| --- | --- | --- | --- | --- | --- | --- |
|  | Beta-coefficients | Standard error | p-value | Beta-coefficients | Standard error | p-value |
| Variables |  |  |  |  |  |  |
| Age (years) | -0.668 | 0.140 | <0.001 | -0.576 | 0.142 | <0.001 |
| Sex (reference=women) | 14.952 | 4.236 | <0.001 | 11.059 | 3.985 | 0.007 |
| BMI (kg/m^2^) | 0.731 | 0.486 | 0.136 |  |  |  |
| **Comorbidities** |  |  |  |  |  |  |
| Hypertension | -6.420 | 4.382 | 0.146 |  |  |  |
| Diabetes mellitus | -13.903 | 4.396 | 0.002 | -13.443 | 3.809 | <0.001 |
| Liver disease | 6.655 | 8.112 | 0.414 |  |  |  |
| Heart disease | -3.647 | 6.013 | 0.545 |  |  |  |
| Cerebrovascular disease | 2.130 | 5.472 | 0.698 |  |  |  |
| Lung disease | -14.197 | 7.022 | 0.046 | -10.279 | 6.007 | 0.090 |
| Chronic kidney disease | -2.082 | 6.318 | 0.742 |  |  |  |
| Malignancy | 6.439 | 5.276 | 0.225 |  |  |  |
| **Clinical data** |  |  |  |  |  |  |
| Infection focus |  |  |  |  |  |  |
| Gastrointestinal (reference=respiratory) | 3.008 | 5.437 | 0.581 |  |  |  |
| Genitourinary (reference=respiratory) | 6.124 | 6.021 | 0.311 |  |  |  |
| Others (reference=respiratory) | 9.342 | 7.387 | 0.209 |  |  |  |
| SOFA score | -1.128 | 0.655 | 0.088 | -1.372 | 0.567 | 0.017 |
| Septic shock | -4.892 | 4.468 | 0.276 |  |  |  |
| **Laboratory data** |  |  |  |  |  |  |
| Lactate (mmol/L) | -1.130 | 0.795 | 0.158 |  |  |  |
| White blood cell count (*10^3^/μL) | -0.207 | 0.2436 | 0.398 |  |  |  |
| Platelets (*10^3^/μL) | -0.004 | 0.020 | 0.849 |  |  |  |
| Glucose (mg/dL) | -0.014 | 0.013 | 0.274 |  |  |  |
| Creatinine (mg/dL) | 1.326 | 0.834 | 0.115 |  |  |  |
| Total bilirubin (mg/dL) | 0.798 | 1.167 | 0.496 |  |  |  |
| CRP (mg/dL) | -0.352 | 0.238 | 0.143 |  |  |  |
| Procalcitonin (ng/mL) | 0.058 | 0.078 | 0.456 |  |  |  |
| Albumin (g/dL) | 7.537 | 3.378 | 0.028 | 2.863 | 2.979 | 0.339 |

The factors that were significant at a level of 0.1 in the univariable model were entered into multivariable linear regression model.

**Supplementary Table 2.** The result of the univariable Cox proportional hazard model

| Variables | aHR | 95% CI | p-value |
| --- | --- | --- | --- |
| Age (years) | 1.014 | 0.980–1.048 | 0.432 |
| Sex (reference=women) | 0.585 | 0.243–1.513 | 0.233 |
| BMI (kg/m^2^) | 0.919 | 0.830–1.017 | 0.098 |
| **Comorbidities** |  |  |  |
| Hypertension | 1.500 | 0.613–3.669 | 0.375 |
| Diabetes mellitus | 2.708 | 1.106–6.628 | 0.029 |
| Liver disease | 0.000 | 0.000–Inf | 0.998 |
| Heart disease | 1.311 | 0.438–3.921 | 0.628 |
| Cerebrovascular disease | 0.617 | 0.181–2.106 | 0.441 |
| Lung disease | 2.306 | 0.770–6.904 | 0.135 |
| Chronic kidney disease | 2.145 | 0.779–5.905 | 0.140 |
| Malignancy | 1.111 | 0.404–3.057 | 0.839 |
| **Initial vital signs** |  |  |  |
| Systolic blood pressure (mmHg) | 1.001 | 0.987–1.014 | 0.945 |
| Diastolic blood pressure (mmHg) | 1.003 | 0.979–1.028 | 0.790 |
| Heart rate (/min) | 1.001 | 0.984–1.017 | 0.952 |
| Respiratory rate (/min) | 1.106 | 1.039–1.177 | 0.002 |
| Body temperature (℃) | 0.974 | 0.831–1.141 | 0.741 |
| SpO2 (%) | 0.958 | 0.887–1.036 | 0.282 |
| **Clinical data** |  |  |  |
| Infection focus |  |  |  |
| Gastrointestinal (reference=respiratory) | 0.457 | 0.146–1.144 | 0.180 |
| Genitourinary (reference=respiratory) | 0.321 | 0.071–1.446 | 0.140 |
| Others (reference=respiratory) | 0.897 | 0.250–3.212 | 0.868 |
| SOFA score | 1.226 | 1.081–1.390 | 0.002 |
| Septic shock | 4.522 | 1.642–12.451 | 0.004 |
| Mechanical ventilator use | 9.793 | 3.890–13.881 | 0.006 |
| ICU admission | 4.128 | 1.685–10.111 | 0.002 |
| **Laboratory data** |  |  |  |
| Lactate (mmol/L) | 1.210 | 1.108–1.321 | <0.001 |
| White blood cell count (*10^3^/μL) | 1.034 | 0.993–1.076 | 0.101 |
| Platelets (*10^3^/μL) | 1.002 | 0.999–1.006 | 0.214 |
| Glucose (mg/dL) | 1.002 | 1.001–1.004 | 0.008 |
| Creatinine (mg/dL) | 1.091 | 0.966–1.231 | 0.160 |
| Total bilirubin (mg/dL) | 0.616 | 0.336–1.127 | 0.116 |
| CRP (mg/dL) | 1.013 | 0.969–1.059 | 0.577 |
| Procalcitonin (ng/mL) | 1.010 | 0.997–1.023 | 0.125 |
| Albumin (g/dL) | 0.322 | 0.159–0.652 | 0.002 |
| **Muscle mass of quadriceps femoris** |  |  |  |
| Mean CSA-QF (cm^2^) | 0.961 | 0.937–0.987 | 0.003 |
| Mean right CSA-QF (cm^2^) | 0.960 | 0.936–0.985 | 0.002 |
| Mean left CSA-QF (cm^2^) | 0.964 | 0.939–0.989 | 0.005 |
| Minimum CSA-QF (cm^2^) | 0.959 | 0.933–0.985 | 0.003 |
| Maximum CSA-QF (cm^2^) | 0.965 | 0.943–0.989 | 0.004 |
| Mean dominant leg CSA-QF (cm^2^) | 0.961 | 0.937–0.986 | 0.002 |
| Mean non-dominant leg CSA-QF (cm^2^) | 0.963 | 0.938–0.988 | 0.004 |

Abbreviations: aHR, adjusted hazard ratio; CI, confidence interval; CSA-QF, cross-sectional area of the quadriceps femoris.

**Supplementary Table 3.** The result of the multivariable Cox proportional hazard model (per 10cm^2^ of CSA-QFs).

|  | Multivariable Cox proportional hazard model | | | Stepwise backward elimination model | | |
| --- | --- | --- | --- | --- | --- | --- |
| **Variable of interest** | aHR | 95% CI | p-value | aHR | 95% CI | p-value |
| Mean CSA-QF (per 10cm^2^) | 0.716 | 0.534–0.961 | 0.026 | 0.691 | 0.520–0.919 | 0.011 |
| Mean right CSA-QF (per 10cm^2^) | 0.721 | 0.546–0.952 | 0.021 | 0.704 | 0.536–0.924 | 0.011 |
| Mean left CSA-QF (per 10cm^2^) | 0.723 | 0.534–0.979 | 0.036 | 0.689 | 0.514–0.923 | 0.013 |
| Minimum CSA-QF (per 10cm^2^) | 0.711 | 0.520–0.973 | 0.033 | 0.686 | 0.506–0.931 | 0.015 |
| Maximum CSA-QF (per 10cm^2^) | 0.731 | 0.556–0.961 | 0.025 | 0.706 | 0.541–0.921 | 0.010 |
| Mean dominant CSA-QF (per 10cm^2^) | 0.720 | 0.544–0.954 | 0.022 | 0.705 | 0.536–0.926 | 0.012 |
| Mean non-dominant CSA-QF (per 10cm^2^) | 0.724 | 0.536–0.977 | 0.035 | 0.687 | 0.514–0.920 | 0.012 |

Age, sex, septic shock status, BMI, DM, lactate, glucose, albumin, and SOFA score were adjusted in the multivariable Cox proportional hazard model.

Abbreviations: aHR, adjusted hazard ratio; CI, confidence interval; CSA-QF, cross-sectional area of the quadriceps femoris.

**Supplementary Table 4.** Outcomes according to optimal cutoff of mean CSA-QF.

| Outcomes | High quadriceps femoris mass  (n=49) | Low quadriceps femoris mass  (n=63) | p-value |
| --- | --- | --- | --- |
| 7-day mortality | 1 (2.0%) | 10 (15.9%) | 0.034 |
| 14-day mortality | 2 (4.1%) | 16 (25.4%) | 0.005 |
| 28-day mortality | 2 (4.1%) | 18 (28.6%) | 0.002 |
| Mechanical ventilator use within 24 h | 3 (6.1%) | 21 (33.3%) | 0.001 |

Data are expressed as numbers (%).

Abbreviations: CSA-QF, cross sectional area of quadriceps femoris.

**Supplementary Table 5.** The result of receiver operating characteristic curve of other variables on 28-day mortality.

| **Variables** | AUROC | 95% CI | p-value * |
| --- | --- | --- | --- |
| Lactate | 0.761 | 0.635–0.887 | 0.641 |
| SOFA score | 0.704 | 0.579–0.830 | 0.825 |

*p-value for comparison between AUROC of the variable and AUROC of mean CSA-QF, after Bonferroni corrections

Abbreviations: AUROC, area under receiver operating characteristic curve; CI, confidence interval; CSA-QF, cross-sectional area of the quadriceps femoris.

**Supplementary Table 6.** Factors associated with muscle mass of quadriceps femoris using linear regression model including other nutrition factor.

|  | Multivariable model | | |
| --- | --- | --- | --- |
|  | Beta-coefficients | Standard error | p-value |
| Variables |  |  |  |
| Age | -0.626 | 0.156 | <0.001 |
| Sex (reference=women) | 7.541 | 4.456 | 0.094 |
| BMI | 0.418 | 0.443 | 0.348 |
| Diabetes mellitus | -12.082 | 4.003 | 0.003 |
| Lung disease | -13.036 | 6.242 | 0.040 |
| **Nutritional Factor** |  |  |  |
| Albumin | 5.162 | 3.367 | 0.129 |
| Total lymphocyte count | -2.909 | 1.919 | 0.133 |
| Blood urea nitrogen | -0.016 | 0.099 | 0.867 |
| Creatinine | 0.541 | 0.947 | 0.569 |

**Supplementary Figure 1.** **Examples of measurement of quadriceps femoris mass using panoramic mode.** The yellow scale on the skin was marked every 1 cm, with a slightly larger scale indicated every 5 cm. The cross-sectional areas of quadriceps femoris are areas surrounded by yellow dashed lines. Red double-headed arrows represent thigh muscle thickness, as in previous studies. Purple asterisks represent subcutaneous fat of thigh. Abbreviations: RF = rectus femoris, VM = vastus medialis, VI = vastus intermedius, VL = vastus lateralis, V = vessels (femoral artery and vein), and F = femur.

(A) The cross-sectional area of quadriceps femoris was 70.75cm^2^.


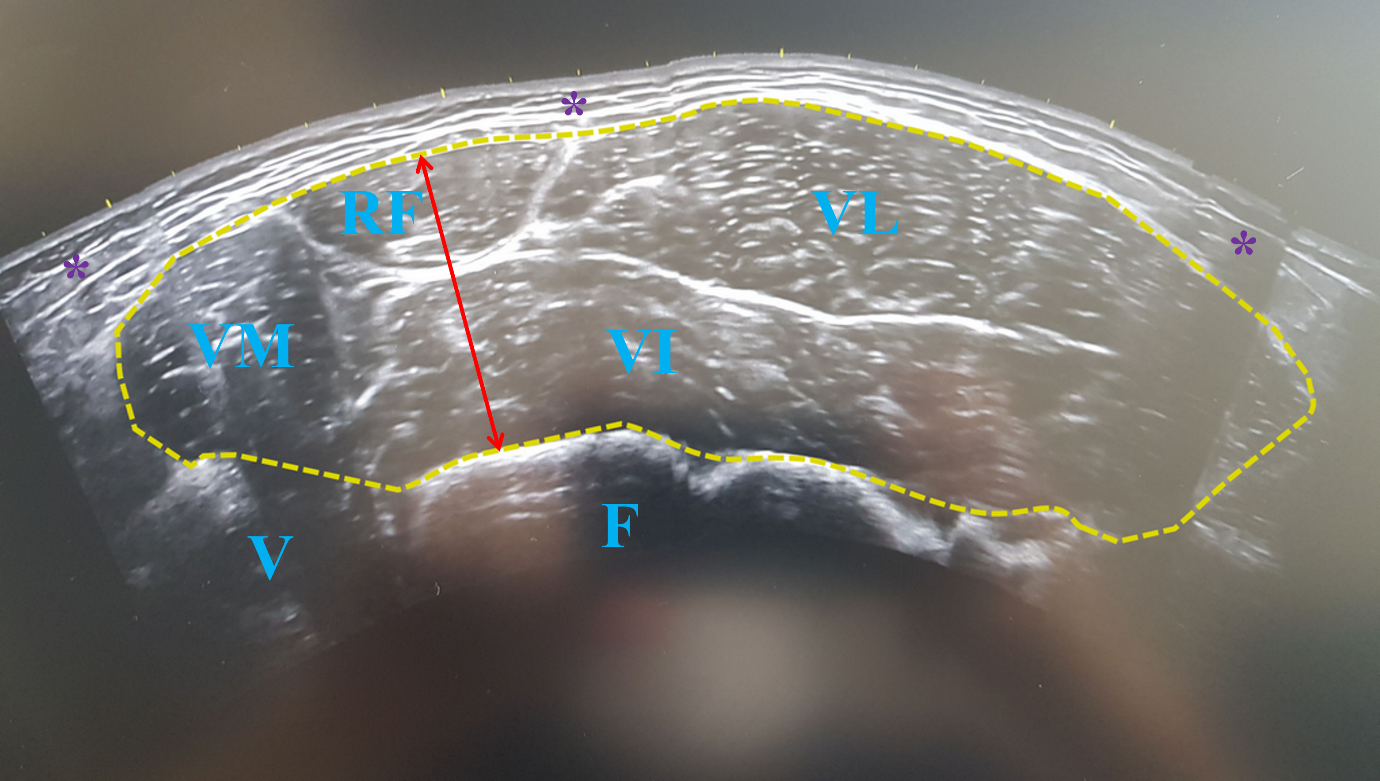


(B) The cross-sectional area of quadriceps femoris was 19.35cm^2^.


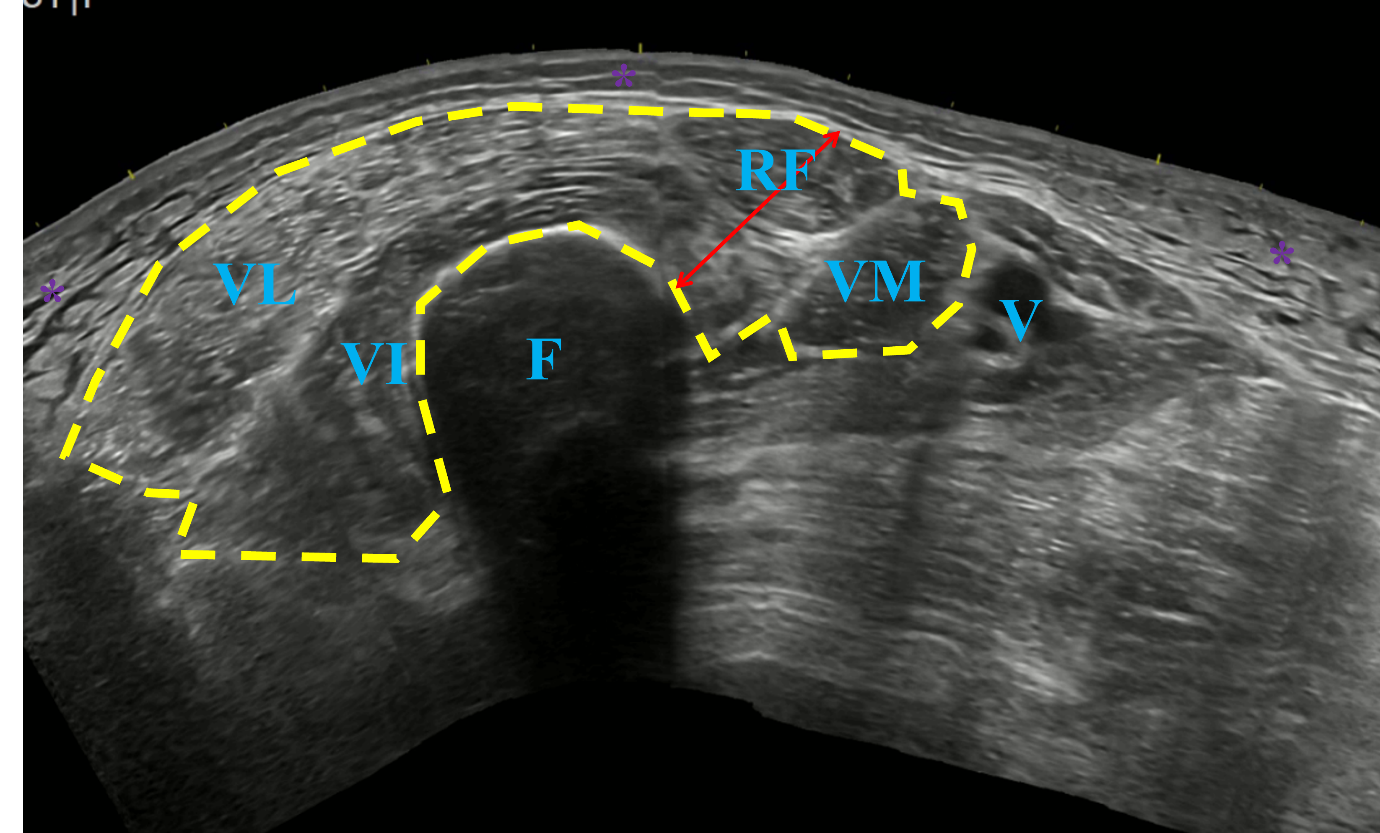


(C) The cross-sectional area of quadriceps femoris was 43.60cm^2^.


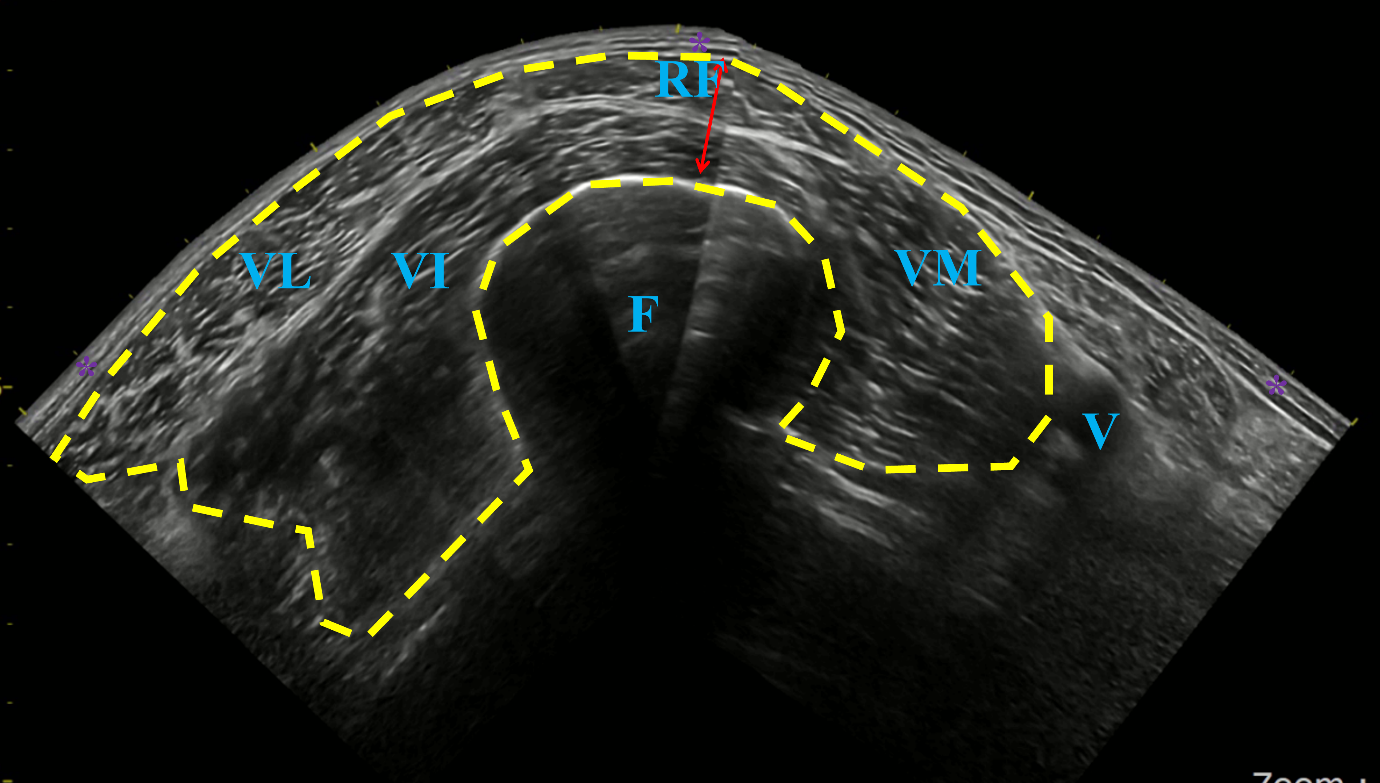


(D) The cross-sectional area of quadriceps femoris was 29.41cm^2^.


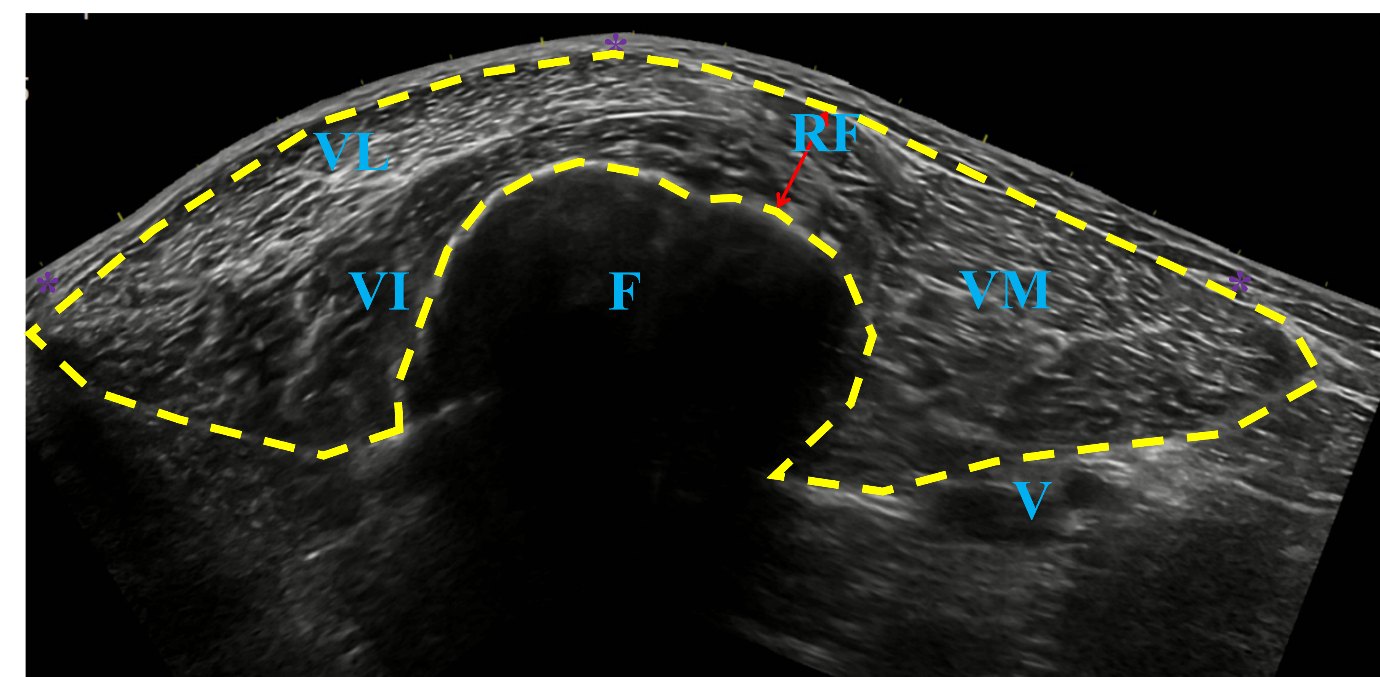

Supplement: Supplementary file 1 — Supplementary Information. [file 41598_2024_63769_MOESM1_ESM.docx]
